# SemNet: Using Local Features to Navigate the Biomedical Concept Graph

**DOI:** 10.3389/fbioe.2019.00156

**Published:** 2019-07-03

**Authors:** Andrew R. Sedler, Cassie S. Mitchell

**Affiliations:** Laboratory for Pathology Dynamics, Department of Biomedical Engineering, Georgia Institute of Technology, Emory University School of Medicine, Atlanta, GA, United States

**Keywords:** literature based discovery, unsupervised learning, text mining, natural language processing, Python

## Abstract

Literature-Based Discovery (LBD) aims to connect scientists across silos by assembling models of the literature to reveal previously hidden connections. Unfortunately, LBD systems have been unable to achieve user adoption on a large scale. This work develops opens source software in Python to convert a database of semantic predications of all of PubMed's 27.9 million indexed abstracts into a semantic inference network and biomedical concept graph in Neo4j. The developed software, called SemNet, queries a modified version of the publicly available SemMedDB and computes feature vectors on source-target pairs. Each unique United Medical Language System (UMLS) concept is represented as a node and each predication as an edge. Each node is assigned one of 132 node labels (e.g., Amino Acid, Peptide, or Protein (AAPP); Gene or Genome (GG); etc.) and each edge is labeled with one of 58 predications (e.g. treats, causes, inhibits, etc.). SemNet computes a single feature value for each metapath, or sequence of node types, between a source node and user-specified target node(s). Several different types of metapath-based features (count, degree weighted path count, and HeteSim metric) are computed and vectorized. SemNet employs an unsupervised learning algorithm for rank aggregation (ULARA) to rank identified source nodes that are most relevant to the user-specified target nodes(s). Statistical analysis of correlation among identified source nodes or resultant literature network features are used to identify patterns that can guide future research. Analysis of high residual nodes is used to compare and contrast SemNet rankings between different targets of interest. An example SemNet use case is presented to assess “the differential impact of smoking on cognition in males and females” using the following target nodes: nicotine, learning, memory, tetrahydrocannabinol (THC), cigarette smoke, X chromosome, and Y chromosome. Detailed rankings are discussed. Overall results suggest a hypothesis where smoking negatively impacts cognition to a greater extent in females, but smoking has stronger cardiovascular impacts in males. In summary, SemNet provides an adoptable method for efficient LBD of PubMed that extends beyond omics-only relationships to true multi-scalar connections that can provide actionable insight for predictive medicine, research prioritization, and clinical care.

## Introduction

Biomedical literature represents an ever-growing repository of complex and interrelated knowledge. PubMed, the largest and most widely used database and search engine, contains over 27.9 million abstracts and counting. Thus, even with the great power of user-specified PubMed searches, it is difficult for a scientist or clinician to keep up with literature in their specialty niche, much less understand the thousands of articles inter-connected to their general domain. Yet, the ability to visualize and evaluate the relative importance of these thousands of literature relationships could be the key to unlocking new etiological or treatment discoveries. In fact, the National Library of Medicine has argued that better knowledge management tools have the potential to impact the efficacy of biomedical research at the level of researchers, policymakers, and scientific publishers (Kilicoglu, 2017). The open-source technology developed here, SemNet, makes PubMed relationship literature mining adoptable by a much greater audience of scientists or clinicians that desire to leverage the power of literature mining to guide their research and development efforts.

To understand the future of literature mining and what SemNet has to offer, it is important to first briefly walk through the history of biomedical literature mining and current limitations. The most recognized forms of traditional literature mining are meta-analysis and systematic reviews (Graves et al., 1996). Data is systematically searched, aggregated, and analyzed. Such analyses compile smaller studies into a larger sample size in order to assess aggregate findings. These studies have been particularly valuable in epidemiology (Berry et al., 2012), clinical medicine decision-making (Park and Han, 2018), as well as narrowly defined experimental examination of multi-factorial diseases (Foley et al., 2015; Bond et al., 2018; Huber et al., 2018). While meta-analysis and systematic reviews continue to be helpful for examining overarching ideas and hypotheses, they have well known limitations. Namely, traditional meta-analyses and systematic reviews only use a very tiny fraction of the literature corpus and require that studies have high degrees of similarity for analytic inclusion. Moreover, such studies typically only examine literature within a niche domain, thus excluding numerous broader relationships that could greatly impact results.

Another historical form of literature mining is the manually or semi-manually constructed “field map,” which visually represents basic concepts within a particular domain (Kim et al., 2016). Field maps employ a user-specified ontology or hierarchy to organize and display literature concepts. While field maps typically utilize article counts, thereby enabling greater literature corpus sizes to be included, they do not capture dynamic features, relationships, or employ mathematical representations that enable ranking or prioritization. Thus, while field maps greatly assist in visually understanding the makeup of a biomedical domain, they do not provide explicitly actionable insight. Finally, traditional field maps are limited to examination of the internal confines of a single and typically narrowly defined domain.

The ability to truly compile the entire scientific literature and use its numerous intertwined relationships within and between multiple domains presents a challenging but very rewarding possibility. It was with this lofty goal in mind that Literature-Based Discovery (LBD) was founded. The field of LBD attempts to capture knowledge from biomedical text and integrate it in a way that makes discovery of new knowledge possible. A common example is Swanson's ABC co-occurrence model, where “A” implies “B” and “B” implies “C” are explicitly found in the literature. The implicit knowledge that “A” implies “C” is used to generate new and actionable hypotheses. Using this co-occurrence inference method, Swanson famously discovered the association between fish oil, blood viscosity, and Raynaud's disease (Swanson, 1986). Thus, fish oil, which reduces whole blood viscosity, is used to prophylactically ameliorate Raynaud's disease—a phasic condition where temporary spastic constriction of arteries reduces bloodflow, most commonly in the fingers or toes, resulting in the digits turning white until arterial constriction abates.

The first step in LBD is to construct a model of the connections between concepts (i.e., genes, proteins, diseases, etc.) in the literature. Concept-based models are visualized in a graphical format called a “biomedical concept graph.” To do this, terminology and methodology from graph theory and social network analysis is borrowed. Biomedical concepts become “nodes” (gene, protein, disease, etc.) and relationships between concepts (inhibits, treats, causes, etc.) become “edges.” Nodes are standardly depicted as points and edges as lines on a graph that, collectively, depict the resultant “network”. The sequence of nodes and edges between two points of interest is defined as a “metapath.” The two key points of interest are standardly referred to as the “source” and the “target” and represent the beginning and end of a metapath, respectively. There can be multiple different metapath types and sequences that encode information, which can be assessed for relevant context. Graphical network information for nodes, edges, and metapaths are mathematically represented as matrices. A weighting system is employed to perform calculations based on frequency and/or edge strength. In modern biomedical LBD systems, the specific type of graphical network model methodologies employed are typically co-occurrence, semantic, or distributional models (Henry and McInnes, 2017).

Notably, most modern biomedical concept graphs have largely focused on gene-gene and gene-protein network assessment. A prominent example is the Tukuru Event Extraction System (TEES) (Bjorne and Salakoski, 2011), which extracts biomolecular events from abstracts of PubMed and from full text articles in PubMed Central; the corresponding database, EVEX (van Landeghem et al., 2013), contains ~40 million events. A variety of other more general literature discovery browsers have also been developed (Hristovski et al., 2006; Smalheiser et al., 2006; Tsuruoka et al., 2008; Cairelli et al., 2013; Poon et al., 2014; Preiss and Stevenson, 2018). Unfortunately, a prevailing theme is that the majority of these modern LBD systems are either too domain-specific (e.g., genomics or proteomics) or they involve a significant amount of effort on the part of the investigator, with relatively limited depth of insight in return. These are key reasons why LBD has been criticized for lack of adoption over the last 30 years (Kilicoglu, 2017).

The present study contends biomedical concept graphs have the potential to be developed into a generalized biomedical literature network that can simultaneously assess etiology, epidemiology, diagnostics, prognostics, or treatment. Moreover, with the power of machine learning, the biomedical concept graph can be dynamically updated and mathematically evaluated to rank key concepts or relationships. Thus, the ability to truly navigate the biomedical concept graph can provide dynamic and actionable insight for predictive medicine, research prioritization, and clinical care. Most of the previous work in this area has been related to drug discovery (Sang et al., 2018), drug repurposing (Himmelstein and Baranzini, 2015), and adverse drug event prediction (Deftereos et al., 2011). However, SemNet extends the power of biomedical concept graphs beyond these aforementioned standard use cases to nearly any research topic or problem, and it can be used for either exploratory or predictive literature analyses.

Therefore, the goals of the present work are to: (1) devise an open-source, adoptable framework for constructing biomedical concept graphs from the popular PubMed and Semantic Medline Databases (SemMedDB); (2) embed a machine learning algorithm for agnostically ranking the importance of features in the biomedical concept graph to a user-specified target (e.g., keyword or group of keywords). This project employs a semantic inference network approach that stores resulting data from a heterogeneous information network. The Python-based software, which we call SemNet, queries a modified version of SemMedDB and computes feature vectors on source-target pairs. The modified version of SemMedDB represents each unique United Medical Language System (UMLS) concept as a node and each predication as an edge. A semantic inference network is constructed in Neo4j using the information contained in SemMedDB. Finally, an unsupervised ranking algorithm uses feature selection to prioritize the “relative importance” of concepts and relationships to the user-specified target(s). In summary, SemNet is able to efficiently derive actionable insight based on *all* of the PubMed literature connections that tie to the user-specified target(s) of interest.

## Methods

As a brief overview, these are the basic workflow and operation steps of SemNet: The first task is to obtain the data for 27.9 million PubMed abstracts needed to concrete the biomedical concept network. We utilize the publicly available SemMedDB and the United Medical Language (UMLS) Metathesaurus, which is explained in more detail below, to convert the raw abstract text into a set of shared categories (e.g., nodes) and relationships (e.g., edges). Using the information contained in SemMedDB, a semantic inference network is created in a Neo4j graphical database. A SemNet user specifies the “target” node(s) of interest that describe the research area or question to be addressed. Once the target is specified, the graphical database is queried to determine the metapaths [e.g., unique sequences of node types] between user-specified targets and identified neighboring source nodes. SemNet then computes three different types of features based on identified metapath patterns and frequencies, and the resultant feature values are vectorized for subsequent analysis. Finally, an unsupervised machine learning algorithm is used to rank identified source nodes based on their relative importance to the user-specified target node(s). Finally, post-simulation clustering and residual methods are used to highlight concepts or regions of interest that are most pertinent to the research domain or question being assessed.

### Creating the Concept Network

Fortunately, there are already excellent resources available that regularly convert and standardize the concepts contained within PubMed literature abstracts. The United Medical Language System (UMLS) Metathesaurus is a large thesaurus of biomedical terms taken from a variety of source vocabularies (Bodenreider, 2004). The most recent release contains 3.85 million concepts and 14.6 million unique concept names from 210 source vocabularies. One significant result of such standardization has been the development of algorithms for identifying mentions of these concepts within the text of biomedical articles, a notable example of which is the MetaMap project (Aronson, 2001). These algorithms have been extended to extract semantic predications from the text, consisting of subject-object-relationship triples (Rindflesch and Fiszman, 2003). The Semantic Medline database (SemMedDB) is a repository of semantic predications extracted from the abstracts of biomedical articles from PubMed using SemRep (Rindflesch and Fiszman, 2003). Each predication ties together two concepts with a specific relationship. Each concept is a unique member of the UMLS Metathesaurus and each relationship is a unique member of the UMLS Semantic Network. In SemMedDB, each node is assigned one of 132 node labels (e.g., Amino Acid, Peptide, or Protein; Gene or Genome; etc.) and each edge is labeled with one of the 58 predications (e.g., TREATS, CAUSES, INHIBITS, etc.). The database is created by regularly processing the abstracts of biomedical articles from PubMed and identifying these concepts and relationships in the text (Rindflesch and Fiszman, 2003). SemMedDB (Kilicoglu et al., 2012) is a repository of nearly 100 million such predications. In the work presented, we utilize the data contained in SemMedDB as of December 31, 2017.

SemNet transforms tabular data into a graph to allow better handling of relationships and faster neighbor node queries. Essentially, SemMedDB is converted into a semantic inference network where resultant data is stored as a heterogeneous information network. Each unique biomedical concept is represented as a node and each unique relationship as an edge. The number of times a given relationship was found in the literature is measured as its edge weight, and the unique article identifiers (e.g., the PubMed IDs) of the abstracts containing the relationship are a property of the edge. A given concept is occasionally classified with more than one type. Node types and node counts are tracked for each node. However, only the most commonly extracted type is used for computational purposes. In the case of a tie, the first identified type is used. This process results in nearly 300,000 nodes and 20,000,000 edges in the final graphical network. The graph is stored in a Neo4j graphical database, which provides fast and efficient queries through the Cypher query language. The database is queried in SemNet using the py2neo Python package. The remaining matrix computation is carried out using custom functions written in Python.

### Computing Metapath-Based Features

The primary objective of the present work was to assess the relationships between nodes, thereby directing user attention to unknown but relevant concepts. Thus, analysis centers on the nodes and edges that connect user-identified concepts (e.g., target nodes) with potential new concepts of interest (e.g., source nodes). To this end, each source node is characterized by the patterns of relationships and node types, or metapaths, that connect it to the target node (e.g., PharmacologicSubstance [TREATS] >Finding [COEXISTS WITH] > DiseaseOrSyndrome). Because a metapath only represents a sequence of types between the nodes, each one is associated with a number of real paths in the network. To extend the given example, there may be several findings that are treated by the pharmacologic substance and coexist with the disease or syndrome.

Several different features can be computed based on these paths, each of which has different properties. The first feature is a simple count of the paths associated with each metapath. The second feature is a degree-weighted path count (DWPC) that simply down-weights paths through highly connected nodes (Himmelstein and Baranzini, 2015). Basically, the degree-weighted path count is used to assess the prevalence of a specific type of path between nodes; the DWPC addresses the number of paths between a source and target node for a given metapath while weighting the connectivity (node degrees) along the path. The third and final feature is the HeteSim metric, a widely accepted measure of similarity in heterogeneous information networks (Shi et al., 2014). Different from homogeneous networks, the paths in heterogeneous networks have semantics, which makes the relatedness of object pair depend on the given relevance path. Following the basic idea that similar objects are related to similar objects, the HeteSim metric proposes a path-based relevance measure. Using terminology from the HeteSim publication (Shi et al., 2014), given a relevance path, *P*, the Hetesim score between two similar objects (nodes), *s* and *t*, (*s* ∈ *R*_1·_*S and s* ∈ *R*_1·_*T*) is:

$$\begin{array}{l} HeteSim\left(s,t\right)|R_{1}\;\circ R_{2}\circ \ldots \circ R_{l}=\frac{1}{\left|O\left(s|R_{1}\right)\right|\left|I\left(t|R_{l}\right)\right|}\sum_{i=1}^{\left|O(s|R_{1})\right|}\ldots .\\ \;\sum_{j=1}^{\left|I(t|R_{l})\right|}HeteSim\left(O_{i},(s|R_{1}\right),I_{j}(t|R_{l})|R_{2}\circ \ldots \circ R_{l-1} \end{array}$$

where *O*(*s*|*R*_1_) is the out-neighbors of *s* based on relation R_1_, and *I*(*t*|*R*_*l*_) is the in-neighbors of *t* based on relation on R_*l*_. Note that a separate equation is defined for two same typed objects, *s* and *t*, that only possess self-relation, *I*: *HeteSim* (*s, t*|*I*) = δ (*s, t* ).

Computation of HeteSim(*s,t|*P) requires iterating over all pairs (*O*_*i*_(*s*|*R*_1_), *I*_*j*_(*t*|*R*_*l*_)) of (*s,t*) along the path (*s* along the path and *t* against the path), and summing up the relatedness of these pairs (Shi et al., 2014). Then, it is normalized by the total number of out-neighbors of *s* and the in-neighbors of *t*. The relatedness between *s* and *t* is the average relatedness between the out-neighbors of *s* and the in-neighbors of *t*. The process continues until *s* and *t* meet along the path. HeteSim(*s, t*|P) measures how likely *s* and *t* will meet at the same node when *s* follows along the path and *t* goes against the path (Shi et al., 2014). For more mathematical details and situational examples for the HeteSim metric, please refer to the HeteSim publication (Shi et al., 2014).

Previous work shows that HeteSim can effectively and efficiently evaluate heterogeneous objects and outperforms other similarity metrics (Shi et al., 2014). In terms of HeteSim's specific use in SemNet, the HeteSim metric provides a powerful metapath feature representation for network analysis and ranking. HeteSim is typically much better for ranking importance of source nodes and metapaths with respect to a user-specified target because HeteSim is not overly biased by sheer counts. Thus, HeteSim was a straightforward choice for inclusion in SemNet and should be the primary feature when analyzing SemNet results for most SemNet use cases.

**Figure 1:**
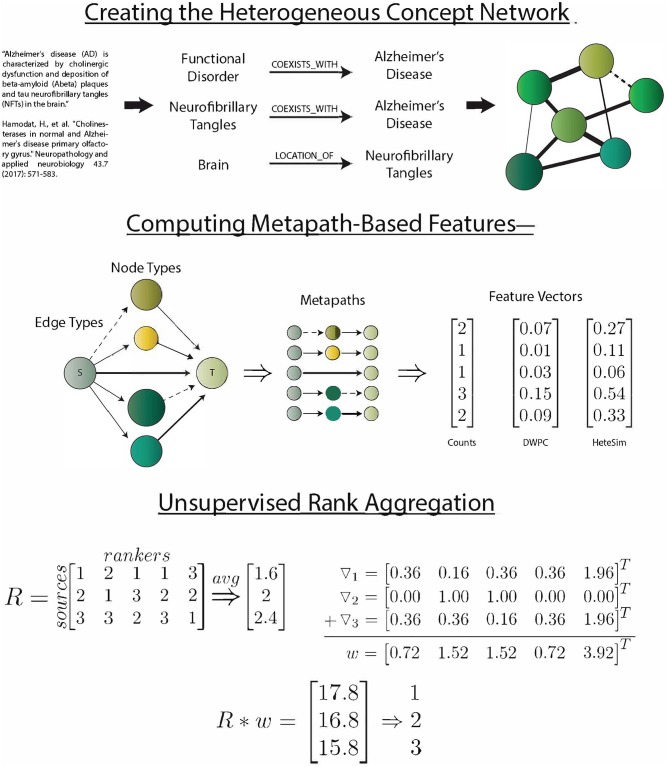
SemNet: a visual overview of the key methods used to create the network, compute metapath-based features, and aggregate rankers. The heterogeneous information network was extracted by running the SemRep predication extraction algorithm on all abstracts in PubMed. Three different metapath-based features were calculated in order to vectorize the complex connections between source and target nodes. Finally, an aggregate ranking scheme (Klementiev et al., 2007) was used to combine feature information into a useful ranking of source nodes with respect to target nodes.

SemNet simulation commences by identifying the source and target nodes of interest. Target nodes, *T*, can be best selected by the SemNet user by searching through the UMLS Metathesaurus and selecting several well-connected nodes relevant to the topic of interest. Typically, the SemNet target nodes will be similar if not literally identical to the keyword(s) the user would enter for a standard PubMed query in their domain of interest. From this starting point, SemNet's constructed graph database in Neo4j is queried to find the set of immediate neighbor nodes. The identified immediate neighbor(s) become the source nodes, *S*. The method of finding *S* should be customized to the specific application. For example, in an exploration of the connections between smoking and performance on a memory test, we set *S* to be all of the nodes that are directly connected to (Learning *OR* Memory)AND(THC *OR* Cigarettesmoke *OR* Nicotine). In the prior example, one of the target nodes of is interest is cigarette smoke. It is not a requirement that the source nodes of interest directly connect to the nodes comprising *T*, but starting at *T* is one straightforward way to define *S*. Once *T* and *S* are defined, the three metapath-based features are computed (count, DWPC, and HeteSim) for all metapaths of two or fewer edges that relate each node in *S* to each node in *T*. For this network, considering paths longer than 2 was intractable, but it could be done with large computational resources if post-analysis of initial simulations deems it appropriate. The procedure results in an *s* x *t* x *m* x *f* matrix, *X*, where *s* is the number of sources, *t* is the number of targets, *m* is the number of metapaths, and *f* is the number of features. Figure 1 visually summarizes the processes of SemNet.

### Source Node Clustering

Once connections between source and target nodes have been vectorized, it is trivial to compare the relationships of two source nodes with a given target node. By computing the cosine similarities of the feature vectors, the similarity of the source-target relationships are computed. The result is an *s* x *s* similarity matrix, which can be hierarchically sorted to produce an intuitive map of the similarities of connections with the target node. Specifically, this work used the scipy and seaborn implementations (Oliphant, 2007; Waskom et al., 2014).

### Unsupervised Rank Aggregation

Modern internet search engines have proven that some mechanism for ranking search results is crucial for usability. Likewise, in the case of semantic inference networks, there is a need to rank identified source node “importance” to user-specified target nodes. However, “importance” of a specific node can change based on the context of the search. Thus, there is not one unique nodal ranking but rather a collection of rankings for each node with respect to each target for each feature. SemNet employs an unsupervised learning algorithm for rank aggregation (ULARA) that is based on a linear combination of ranking functions guided by the principle that the relative contribution of an individual ordering to a joint ranking is determined by its tendency to agree with other members of the expert pool (Klementiev et al., 2007). In brief, the ULARA method derives a surrogate signal in the absence of labeled data, referred to as an incidental supervision signal, which is based on the agreement of a given ranker compared to the plurality of the other rankers. ULARA is chosen for the SemNet application because it is one of the only methods capable of performing parameterized rank aggregation without the need for supervision. As such, ULARA is a popular method for information retrieval and data fusion problems, such as when retrieved documents from a large online corpus are ranked for a given query. Gradient descent is used to optimize ranking weights.

The main equation of ULARA (Klementiev et al., 2007) is illustrated below. The right hand side of the equation is equal to the variance-like measure, which is used to measure ranker agreement. For this initial short explanation, we will keep consistent with previously published nomenclature and equations (Klementiev et al., 2007). ULARA takes a set of queries, *Q*, of which we do not know the true ranking. For each item, *x*, and query, *q*, the expert ranking for the *N* rankers is determined using r_i_(q,x); the mean (μ(q,x)) is calculated; the gradient is determined; and the weight update is made. Once the weight updates are completed, the weight vector is normalized to generate a probability vector for evaluation. The original ULARA in Klementiev et al. (2007) provides both additive and exponential learning rate algorithms for gradient descent. SemNet employs the additive algorithm code shown in the original Klimentiev study (Klementiev et al., 2007). For greater details on the mathematical details, performance, and pseudocode explanation specific to ULARA development and implementation, please refer to the previously published work (Klementiev et al., 2007).

$$\begin{array}{l} \nabla_{i}=\frac{\partial \delta_{i}\left(q,x\right)}{\partial w_{i}}={\left[r_{i}\left(q,x\right)-\mu \left(q,x\right)\right]}^{2} \end{array}$$

Putting the above original explanation of ULARA into context: “Documental retrieval and ranking” is equivalent to source node retrieval and ranking in SemNet, with SemNet's semantic inference network constructed from SemMedDB serving as the corpus. ULARA is used to rank identified source nodes for a given set of user-specified target nodes. Each metapath-based feature is considered a noisy ranker, where a higher feature score means a higher rank. As such, rankers can be aggregated by learning weights by employing the principle that good rankers correlate well with the across-ranker consensus for a given source (Klementiev et al., 2007). The feature matrix can be converted to a ranking matrix, *R*, by assigning ranks along the *s* dimension. In the case of the count features, where many sources may have the same feature value, a dense ranking algorithm is utilized. We utilize the built-in Python PANDAs method, Pandas.Series.rank (method = “dense”, ascending = False). When ranking with respect to two target nodes simultaneously, more rankers can be added by concatenating along the *m* dimension to include features with respect to multiple nodes in *T*. Using the previously described gradient descent algorithm that finds optimal ranker weights based on agreement with the mean ranking for each source node, each source is re-ranked with respect to each target for each feature type. If there are two different specified targets, *Z* and *A*, two noisy ranker matrices for *Z* and *A* are developed for each feature type (count, DWPC, HeteSim metric). Each of these is *m* x *n*, where *m* is the number of nodes, *n* is the number of features. The *Z* matrix includes features that connect each node with the *Z* target node, while *A* includes concatenated features connecting each node to the *A* target node(s). Note that the number of features can be different for *Z* and *A* because a different set of metapaths apply in each case. A dense ranking algorithm is used to convert feature values into rankings within each column, and then these are aggregated according to the additive ULARA sub-algorithm given in Klementiev et al. (2007).

### Ranking Correlations

Aggregate rankings with respect to the target nodes are sufficient for exploration of that target node, but often scientists may want to examine similarities and differences across multiple target nodes. At a high level, the target node rankers can be compared based on the similarity of their rankings to the source nodes. Rankers that are *too* similar raise the question of potential bias. The correlation of rankings can be compared with respect to different targets and using different feature types. Pair plots and Kendall's τ (Knight, 1966) ranking correlation are useful for visualization of ranking performance. High correlation across all targets is an indicator of bias, while some correlation and some uncorrelation is to be expected for a superior, less-biased ranker.

### High Residual Nodes

More useful knowledge can be obtained by looking at the specific source nodes that are highly ranked with respect to a given target. Similarities between two target concepts can be judged by examining their average ranking of a given node and comparing their ranking differences by looking at the high-residual source nodes. High-residual nodes are defined as those which are ranked significantly different by the two aggregate rankers.

### Software Package Information

SemNet is written in Python 3.6.4. The following Python libraries/packages were utilized: Hetio 0.2.8 is used for metagraph and metagraph to string operations; Xarray 0.10.7 is used for storing labeled, multidimensional data; Numpy 1.15.0 is used for performing linear algebra operations; Py2Neo 3.1.2 is used for interacting with the Neo4j instance; Pandas 0.23.0 is used for handling 1-D and 2-D data and for the dense ranking algorithim; Sklearn 0.19.1 is used for optimization; Scipy 1.1.0 is used for linear programming and Kendall's τ calculation (Knight, 1966); Matplotlib 2.2.2 is used for plotting line, bar, and scatterplots; Tqdm 4.23.4 is used for progress bar; Seaborn 0.8.1 is used for heatmap visualization.

The tools necessary to perform SemNet analysis are Python (SemNet is written in a downloadable Python package); Neo4j, and a copy of SemMedDB. Neo4j installation/account is required to make the biomedical concept graph (www.neo4j.com). The SemMedDB, which contains the PubMed data used by SemNet, is available for download from the National Library of Medicine and Semantic Medline. The full SemNet code package is available for download on GitHub.

## Results and Discussion

This section includes: a basic walk-through of generalized SemNet results and performance with discussion on how to visualize and optimize SemNet analyses; a detailed example of insight gained for a specific use case for a research question examining “how cigarette smoke or THC differentially impacts learning or memory in males and females” (see Table 1 for SemNet target nodes); a discussion of other general uses for SemNet; and limitations and future directions for SemNet.

**Table 1:** List of target nodes considered and/or used for the presented SemNet use case to “assess the the differential impact of smoking on cognition in men and women.”

**Name**	**Type**	**Identifier**	**Degree**	**# of AAPPs**	**Use**
Impaired cognition	fndg	C0338656	62,483	554	–
Nicotine	hops	C0028040	37,825	1,657	Finding sources and target
Learning	menp	C0023185	14,340	408	Finding sources and target
Memory	menp	C0025260	12,970	678	Finding sources and target
Tetrahydrocannabinol	orch	C0039663	11,058	547	Finding sources and target
Cigarette smoke	hops	C0239059	8,663	720	Finding sources and target
X Chromosome	celc	C0043292	7,770	103	Target
Y Chromosome	celc	C0043381	4,547	369	Target
Mental association	menp	C0004083	1,614	125	–
Learning ability	menp	C0233832	410	24	–
Association cortex	bpoc	C0596129	375	14	–
Learning performance	fndg	C0582590	359	22	–

*The name column is the name of the node; type column is the node type as assigned in SemMedDB; the identifier column is the identifier assigned by SemMedDB; degree represents connectivity; # of AAPPs column represents number of nodes labeled as SemMedDB node type AAPP that are associated with the node name; and use column represents whether the node was used as a user-specified target node, a possible source node, or both*.

### Initial Assessment of the Network and Features

It is tempting to immediately jump to the “relative importance” ranking of identified sources nodes, which is the key deliverable of SemNet. However, performing initial assessment of the network provides important insight, and therefore, should not be skipped. Such understanding helps to put ranking results in perspective, provides key sanity checks, and assesses if/how the SemNet analysis can be further optimized for a specific use case. There are three initial assessments that are recommended: (1) assessment of network connectivity to insure both high overall connectivity as well as diversity of metapaths between source nodes and target nodes; (2) source node clustering to assess hierarchical physiological concepts that could be contributing to source node identification with respect to a specified target; (3) assessment and distribution profiling of metapath-based features.

#### Network Connectivity

The dataset of 27.9 million PubMed indexed article abstracts consisted of 300,000 nodes and 20,000,000 edges, which were stored effectively in a Neo4j database. RAM usage ranged from 5 to 20 GB, depending on query load. Most interaction with the database consisted of sending repeated neighbor queries. When implementing feature calculations using 40 parallel workers, compute times ranged from 10 min to 12 h, depending on the number of sources and targets. Each source/target pair computation averaged 2–3 s with parallel queries. Since it is derived from the literature, the network is obviously biased toward highly referenced concepts. It is important to note that the concept network does not represent a biological system, but is at its heart a model of the literature.

**Figure 2:**
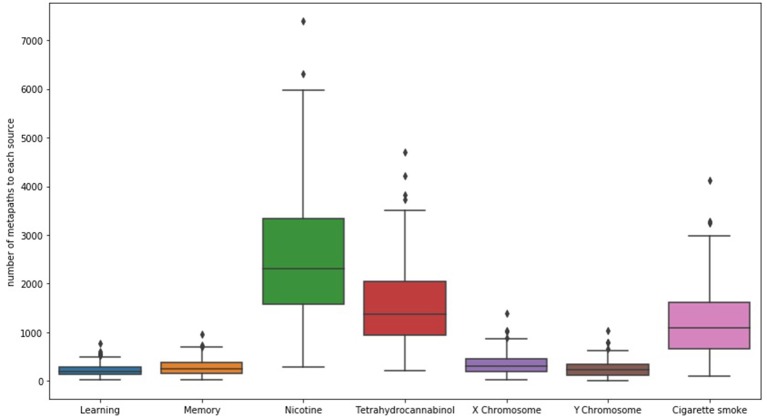
The distribution of the number of unique metapaths between 324 source and 7 target nodes (depicted as bars) for the example SemNet use case, “differential impacts of smoking (cigarette smoke or tetrahydrocannabinol) in men and women.” Nicotine generally had the most diverse connections to the source nodes in this case. The figure shows that number of metapaths varies significantly depending on the target node of interest. Specific and well-known chemicals and biological entities are generally the most highly connected.

When examining SemNet results, a good first step is chart the number of unique methapaths from each identified source to each of the user-specified targets. Examining unique metapath counts helps to better understand network connectivity and network heterogeneity; it can also help aid in further optimizing the selection of relevant target nodes. For example, Figure 2 shows a SemNet analysis with 7 user-specified target nodes (learning, memory, nicotine, tetrahydrocannabinol, X chromosome, Y chromosome, and cigarette smoke); each node is represented by a bar on the graph. The largest bar represents the target node with the most diverse connections to the identified source nodes (“nicotine,” shown in green); nicotine has about 3,500 unique metapath connections to identified source nodes. Tetrahydrocannabinol (or THC, shown in red) and cigarette smoke (shown in pink) also have diverse connections. The connections to learning, memory, and the X and Y chromosomes are much less diverse.

**Figure 3:**
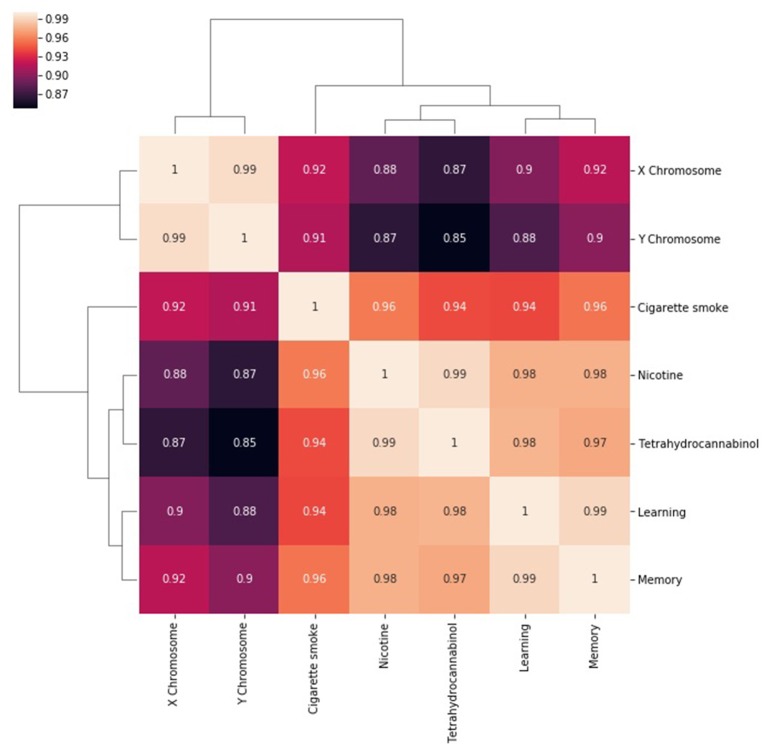
A heatmap of the correlation between the number of metapaths for a given source node with respect to pairs of target nodes. The number of metapaths to the X chromosome target correlates with the number of metapaths to the Y chromosome target, but less so with the other targets. Similar relationships can be seen between nicotine and THC and between learning and memory. Correlations remain generally high because entities that are well researched are likely to be highly connected in general.

The next step in SemNet analysis is to assess the correlation between the number of metapaths for a given source node with respect to pairs of target nodes (Figure 3). That is, what source nodes do pairs of target nodes share? It is expected that correlations will be rather “high” (e.g., >0.85) in highly connected domains where the target nodes are heavily cited. However, there still should be some diversity in the correlations visualized.

High connectivity is always favored for SemNet literature networks. Just like more patients increases clinical trial analytical power, higher connectivity increases the statistical power and robustness of the SemNet network. However, there needs to be a balance between high connectivity and diversity in the source-target pairs in order to obtain the most specific and actionable insight. There is no “one size fits all” solution to say what percentage of connectivity or diversity is required for a given problem. Rather, it will greatly vary based on the target nodes of interest and usage case. Using SemNet to examine different combinations of targets is advisable (e.g., analogous to a model sensitivity analysis) to ascertain a better feel of how connectivity and diversity vary with the user's domain of interest. More general nodes will tend to be more highly connected whereas more specific/detailed nodes will tend to be less connected. However, less connected does not always mean less important. Thus, including some general and some more specific target nodes in the SemNet analysis is a good rule of thumb to balance connectivity and diversity and thus, correspondingly, increase the chance of revealing new, actionable insight.

#### Source Node Clustering

**Figure 4:**
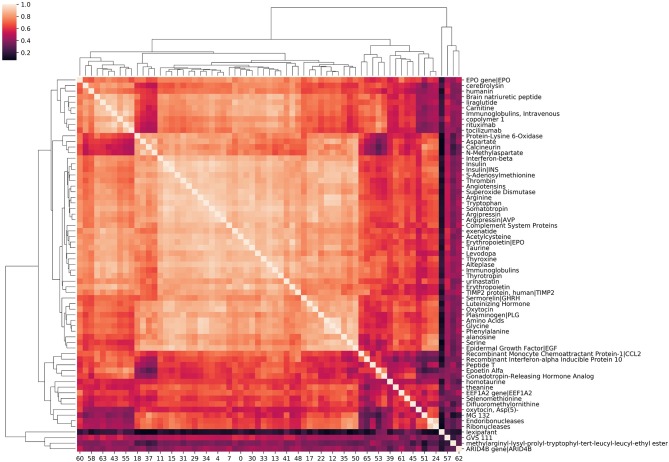
A hierarchical clustering of amino acids, peptides, and proteins associated with “impaired cognition.” The intensity of the heatmap corresponds to the similarity of each concept pair's relationship with impaired cognition. Similarity values are cosine similarities of metapath count features. Examining correlation among source nodes helps the user to assess underlying hierarchical concepts that could be driving the source node selection.

Source node clustering allows a straightforward, agnostic way to examine the similarities between source nodes connected to a specified target node. Visualizing source node clusters enables the user to quickly assess potential aggregate, physiological concepts contributing to source node identification with respect to the target. While this step is not required, it does aid the user in better understanding the network. Figure 4 illustrates clustering of source nodes for a separate SemNet use case where the target node was “impaired cognition.” As seen in Figure 4, biologically similar source nodes tended to cluster together (e.g., amino acids clustered near similar amino acids, etc.). Interestingly, regardless of use case, similarity clustering of source nodes performed much better with count-based metrics than either DWPC or HeteSim. This is likely because: (1) counts are inherently larger values, while DWPC and HeteSim range between zero and one; (2) counts are less reliant on interactions. Again, clustering of concepts does not directly represent biological similarity but similarity in patterns of connections to the target nodes found by SemRep. Clustering can help to determine if user-specified target nodes are resulting in source nodes that appear to be sufficiently relevant to the SemNet use case. For example, if the preponderance of identified source nodes are deemed by the user to be “too general” or appear to cluster around too few physiological concept(s), it may suggest that a revised set of target node(s) should be tried. Finally, source node clustering should not be mistaken for rank aggregation or “importance,” which is a completely separate process, as outlined below.

#### Assessment of Metapath-Based Features

Once network connectivity has been assessed, the next step is to evaluate the three metapath-based feature types (count, DWPC, and HeteSim) used in SemNet. Recall that these three features can be used to rank “important” concepts. Picking the “best” or primary feature for ranking will depend on the specific SemNet use case and target selection. Initial evaluation as outlined below will help put final rankings into context and help assess which feature should be the primary for the use case.

**Figure 5:**
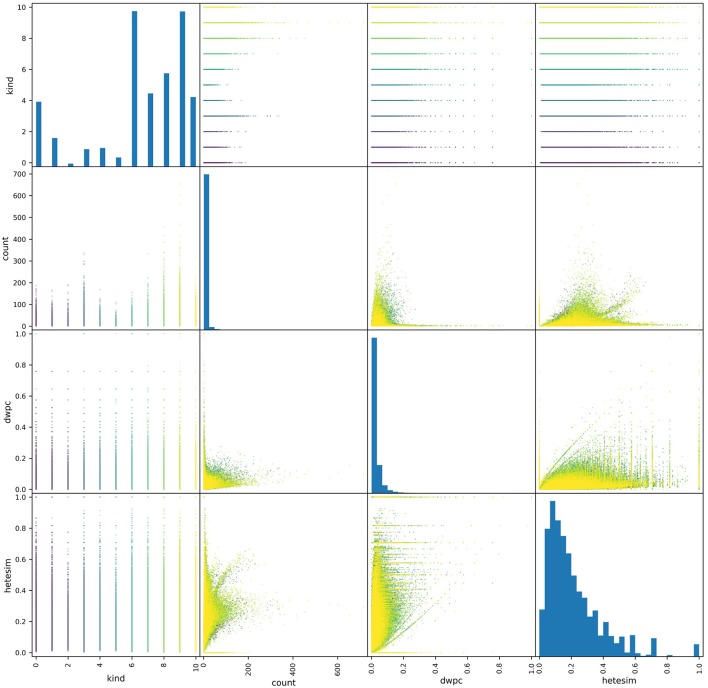
A pair plot showing correlations between metapath-based features across a set of source nodes of different types. All non-zero features were plotted, where each point represents the feature values of a metapath for a given source-target pair. A total of about a half-million points are colored according to their source type. More distribution represents a less-biased ranking feature. Thus, HeteSim, which has the most distribution or “diversity,” is the least biased feature for the use case shown.

One key feature profiling task is to assess the distributions of metapath-based features. Pair plots provide an excellent visual tool for examining the distribution of metapath-based features. As shown in the pair plots of Figure 5, the non-zero occurrences of the three features of interest showed very different distributions. Both count and DWPC metrics tended to skew right, with a majority of values being very close to zero. By contrast, the HeteSim metric was much more evenly distributed across its range. Additionally, the range of the count metric values was highly dependent on source type (specified as “kind” in the figure), while DWPC's and, to a greater extent, HeteSim scores were more uniform across source types. Overall, this perspective informs the use of the network in several ways. First, the bias consequences of using simple count-based features are evident. Some relatively generic source nodes (e.g., Protein, Amino Acid) will have a disproportionately large number of connections and will therefore be judged as important even if they are not actually meaningful. Second, it is evident that DWPC is also heavily skewed, with relatively few values >0.1 in its entire 0-to-1 range. Thus, for this particular use case shown in Figure 5, HeteSim is the most superior feature for ranking “importance” of concepts in a way that minimizes count bias or is source node type (e.g., kind) dependent.

**Figure 6:**
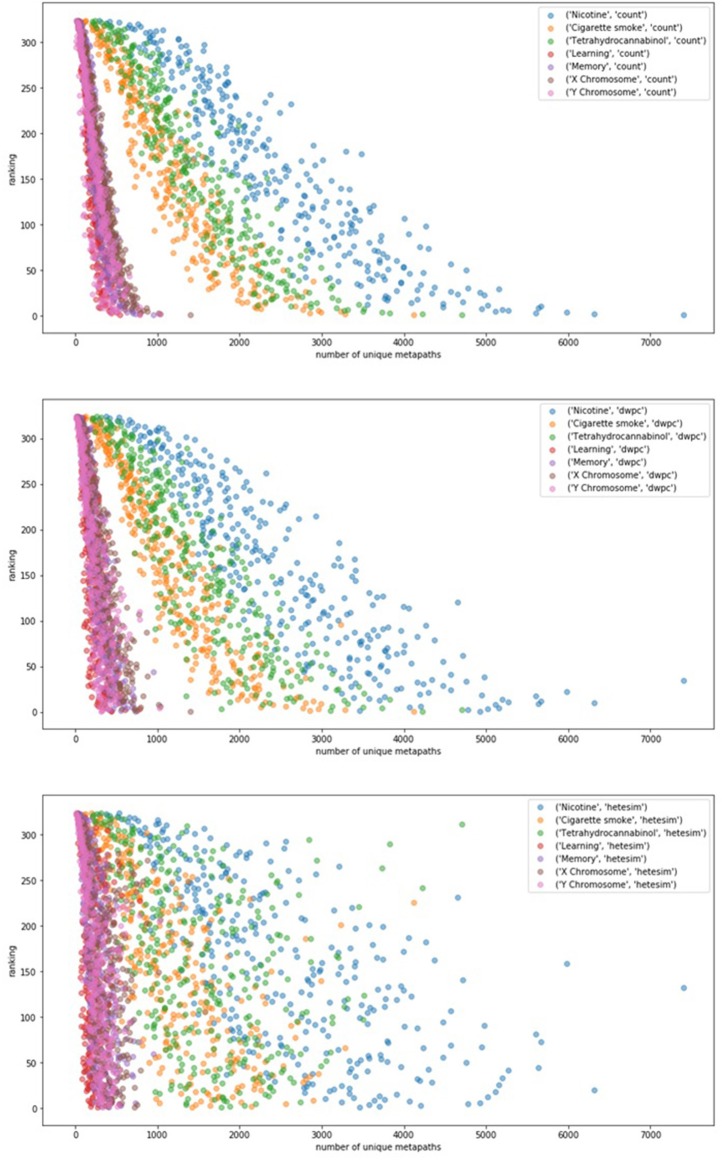
The relationship between number of metapaths and final ranking outcome for different feature types (top: count, middle: DWPC, and bottom: HeteSim) is plotted. The figure contains 324 source nodes with respect to 7 targets using each of the three feature types. Number of metapaths predicts final count and DWPC-based ranking with fairly high accuracy, while the HeteSim rankings appear to be less influenced by the sheer number of non-zero metapath scores. Thus, the HeteSim is a superior ranking because its rankings are less biased toward metapath count.

In an effort to examine potential bias of rankings by the sheer number of metapath-based features, ranking was plotted as a function of unique number of metapaths for each of 7 user-specified target nodes (Figure 6). Figure 6 illustrates that count-based and even DWPC-based rankings tend to rank source nodes higher when they have a larger number of metapaths to the target. While this makes sense, it is intended for the SemNet ranking method to detect more nuanced information about the connections. In order for more nuanced connections to be appropriately ranked as important, a feature ranker must have a way of normalizing for counts. The HeteSim metric, as described in Methods, was invented for this purpose in mind. Fortunately, Figure 6 does affirm that the HeteSim metric does distribute rankings such that there is less correlation between overall ranking and number of unique metapath counts. Admittedly, there is still some remaining bias, as nodes with a higher number of metapaths still tend to be ranked higher even when using the HeteSim feature as the primary ranker. Nonetheless, HeteSim is much less biased than count or DWPC.

Based on both simulations in prior literature examining HeteSim and multiple different use cases specifically examined to date in SemNet, we contend that the HeteSim feature is currently the best in most SemNet use cases. However, feature profiling as outlined above should be repeated for each SemNet use case as there could be specific use cases, depending on target node selection and connectivity, where HeteSim may not be the preferred primary ranking feature. As new rank aggregation features are developed in the future, those could be added or swapped into SemNet (see Limitations).

### Unsupervised Rank Aggregation

**Figure 7:**
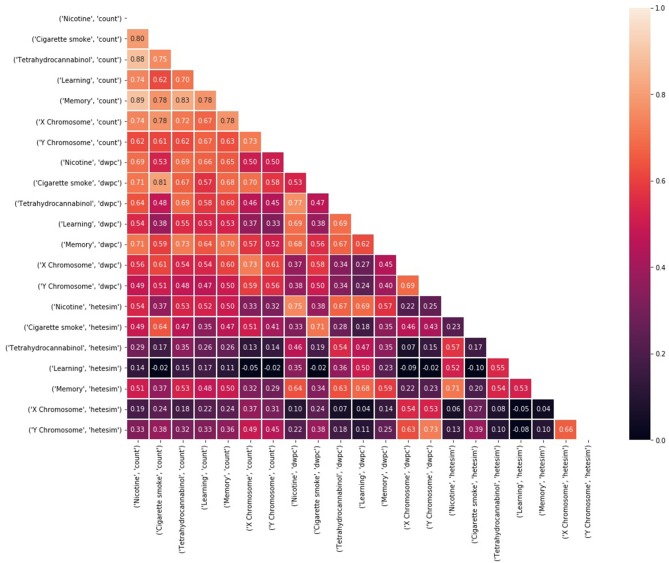
Kendall's τ rank correlations (Knight, 1966) between rankings of 324 source nodes with respect to each of 21 target node and feature type combinations. Rankings are based on a weighted sum of noisy, feature-based rankings, where weights are learned based on consistent agreement with the average ranking.

**Figure 8:**
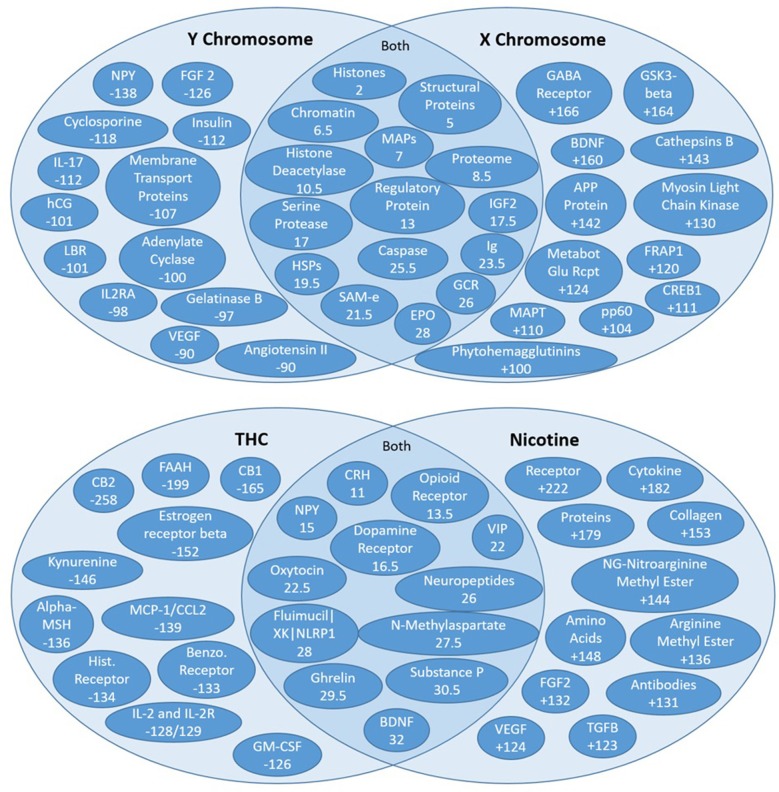
High residual assessment of rankers. Numbers in the overlapping regions represent the highest average ranking between the two target nodes. (Top) Comparing the shared source node rankings between the X and Y chromosomes. The non-overlapping regions of the Venn diagrams contain the nodes with the largest discrepancy between the two rankers (e.g., NPY was ranked 138 spots higher with respect to the Y chromosome than the X chromosome). (Bottom) Comparing the shared source node raking between nicotine and THC.

Unsupervised rank aggregation is arguably the greatest analytical asset of SemNet. Unsupervised rank aggregation enables source nodes to be ranked by “relative importance” with respect to the target node(s) using metapath-based features (count, DWPC, HeteSim). There are two different types of suggested analysis for examining SemNet result rankings. First, standard ranking correlations between source-target pairs provide a straightforward method to quantify, compare, and contrast “relative importance” (Figure 7). Second, high residual node analysis is excellent for comparative analysis between two different targets [or “rankers”] (Figure 8). In particular, residual node analysis greatly simplifies construction of actionable insights.

#### Ranking Correlations

When a set of “rankers” is created with respect to a set of target nodes, it is expected that the targets will rank the source nodes in some unique way. If there is too much similarity between rankings with respect to different source nodes, it is assumed there is an underlying bias in the rankings. Figure 7 compares Kendall's τ (Knight, 1966) correlation between pairs of ranking models based on different targets and metapath-based features. For the results shown in Figure 7, 324 amino acid, peptide, and protein source nodes were ranked with respect to 7 target nodes for each of the three feature types (count, DWPC, Hetesim). Thus, Kendall's τ (Knight, 1966) was computed between 21 different rankers. Notice that ranking correlations are fairly high, even for very different concepts, when using the count feature, but more differentiated when using the DWPC and HeteSim features. These results illustrate once again that rankings by count-based features are biased toward highly connected nodes and metapaths. There is more confidence in the HeteSim rankings because they are more diverse; in fact, there are even some pairs that have nearly zero correlation. Examination of ranking correlations gives a high level view of the overall correlations between targets and features.

#### High Residual Nodes

The residual method is very useful for exploring new concepts that may be distinctly relevant to two different target nodes or “rankers.” Recall that unsupervised rank aggregation determines an aggregate rank order for the source nodes identified for each target. Comparing both the mean and the specific rankings of source nodes shared by two different targets can be extremely informative. The difference in a shared source node ranking between two rankers is called the residual ranking. Residual nodes are best illustrated on a Venn diagram (Figure 8). The highest mean-ranked nodes (e.g., the highest ranked shared source nodes) are placed in the overlapping, middle section of the Venn. The highest residual nodes for each respective target node are placed on each target's respective side of the Venn diagram. Thus, the non-overlapping regions of the Venn diagrams contain the nodes with the largest discrepancy, whereas nodes in the overlapping sections are more similarly ranked between the targets or “rankers.”

Figure 8 illustrates the residual rankings for 324 amino acids, peptides, and proteins with respect to the same 7 target nodes. Rankings with respect to one target node were simply subtracted from rankings with respect to the other target node and sorted by absolute value to find the most significant discrepancies between the two rankers. As noted above, the non-overlapping regions of the Venn diagrams contain the nodes with the largest discrepancy between the two targets (e.g., NPY was ranked 138 spots higher with respect to the Y chromosome than the X Chromosome).

Note that “rankers” and targets can be used interchangeably when discussing the mapping of SemNet high residual nodes. Typically, we think of comparing two different target nodes that were both specified and simultaneously assessed within the same SemNet simulation, as was done in Figure 8. However, the reason for the separate nomenclature of “rankers” is to illustrate that the high residual method can also be used to compare two entirely different SemNet simulations for which the specified target nodes for each simulation are different, but yet both simulations still share many of the same source nodes. The ability to do comparative analysis of say, two different SemNet simulations of two different diseases, and look at both their overlapping and high residual source nodes, is another powerful use case of SemNet. For example, imagine comparing “frontotemporal dementia” with “Alzheimer's disease”. Both are neurological diseases that involve cognitive impairments and share many of the same biological underpinnings, and yet they each are clinically characterized as different diseases. Comparative analysis of the literature for each disease could provide clues for better clinical and etiological differentiation.

### Example Insights From a SemNet Use Case

Below a specific example of a SemNet use case is presented that also aligns with the results figures presented and discussed previously. The use case examined was to “assess the differences between men and women regarding the impact of smoking on cognition.” More specifically, this case study examines differences in the X and Y chromosome connections and differences between cigarette smoking and marijuana (THC) smoking.

#### Defining the Use Case Target Nodes

Table 1 illustrates a list of target nodes searched for a research use case to “assess the differential relationship between men and women of the impact of cigarette smoking or THC (tetrahydrocannabinol) on cognition.” Target node selection is an important process, and should be done iteratively using both expert domain knowledge as well as the SemNet network connectivity-diversity concepts discussed previously. For this case, the final included nodes were: nicotine, learning, memory, tetrahydrocannabinol, cigarette smoke, X chromosome, and Y chromosome. The following nodes were tried but removed from the final SemNet simulation due to low connectivity [connectivity is illustrated by the “degree” column in Table 1]: mental association, learning ability, association cortex, and learning performance. In contrast, “impaired cognition” was initially included but its inclusion produced either too general sources nodes or resulted in many connections to other concepts outside the immediate scope of the research question (e.g., explicit ties to Alzheimer's Disease, aging, etc.). Note that some nodes (nicotine, learning, memory, tetrahydrocannabinol, cigarette smoke) were used as both user-specified target nodes and source nodes; this enabled self-connections to also be included in the network. X and Y chromosome were only used as target nodes in order to minimize extraneous or irrelevant self-connections. “Men or male” or “female or women” could have been used as target nodes to denote gender connections. However, the X and Y chromosome are a better biological representation, and they have more connections to the amino acids, peptides, and proteins (AAPP) node type, which was the selected node type priority for this specific SemNet use case.

#### Initial Assessment of the Use Case Network

Initial assessment for this SemNet use case was performed according to the previous section and Figures 1–6. As previously noted, the HeteSim feature was the best primary ranker, and is used to discuss the final rankings and corresponding context.

#### SemNet Use Case Ranking Analysis

Kendall's τ (Knight, 1966) rank correlations between rankings of 324 amino acid, peptide, and protein (AAPP) source nodes are shown with respect to each of the 21 target node and feature type combinations (Figure 7). The diversity of correlation, especially among the HeteSim features, is again very apparent. Nicotine HeteSim correlates moderately well with THC HeteSim, learning HeteSim, and memory HeteSim. THC HeteSim correlates moderately well with learning and memory HeteSim.

There is negligible correlation with X and Y chromosome and the other 5 target node HeteSim features. Admittedly, these results are at the highest level and provide only general trends and an assessment of SemNet connectivity-diversity, as previously discussed.

The Kendall τ method (Knight, 1966) can be repeated to look deeper into underlying source node clusters for each target. Here, we were most interested in comparing different rankings of AAPPs on the X and Y chromosome in the context of cigarette or THC usage impact on cognition. Differential comparisons are best made using the high residual method.

Figure 8 illustrates the Venn diagram or the high residual node ranking analysis. Figure 8 (top) compares connections to the X and Y chromosomes. The key AAPP source nodes for the Y Chromosome were angiotensin II, vascular endothelial growth factor (VEGF), and neuropeptide Y (NPY). Angiontensin II is a hormone involved in blood pressure. Smoking decreases the ability of the body to protect against ACEI (Angiotensin-Converting Enzyme Inhibition), which leads to increased constriction of blood vessels (Roehm et al., 2017). It is involved in cognitive impairment and even brain injury (Mogi et al., 2012). VEGF is a protein involved in the formation of blood vessels. Cigarette smoking is associated with high VEGF levels, leading to an increase in blood vessel production and repair (Ugur et al., 2018). Cognition is known to increase with an increase in VEGF levels. Both Angiotensin II and VEGF were ranked 90 spots higher in relative importance in the Y chromosome connections compared to X chromosome connections. Neuropeptide Y is an amino acid that plays a pivotal role in cognition and the modulation of homeostasis and neurogenesis (Chen et al., 2007) and is the most abundant neuropeptide in the central nervous system. Cigarette smoking has been found to decrease the levels of NPY in the hypothalamus (Chen et al., 2007). NPY was ranked 138 spots higher in Y Chromosome connections.

Brain Derived Neurotrophic Factor (BDNF) is a protein responsible for maintaining the survival of nerve cells and for regulating synapses. It helps to maintain synaptic plasticity, which contributes to learning and memory. A prior study on smoking and BDNF has demonstrated that nicotine leads to an increase in upregulation of BDNF (Lang et al., 2007). BDNF was found to be ranked 160 spots higher in the X chromosome connections compared to the Y chromosome connections. Metabotropic Glutamate Receptor (MGluRs) are involved in learning, memory and anxiety. They are expressed in pre/post synaptic neurons in the hippocampus, cerebellum and cerebral cortex and have positive effects on cognition (Olive, 2010). Smoking decreases MGluR density (Hulka et al., 2014). MGluRs are ranked 124 spots higher in X chromosome connections compared to Y chromosome connections.

A unique AAPP that was ranked high in both X and Y chromosome connections is erythropoietin (EPO). EPO is a hormone produced by the kidneys that causes the bone marrow to produce more red blood cells and initiates synthesis of hemoglobin. An increase in EPO is known to increase cognition as it pertains strongly to delivery of oxygen to the brain. Smoking has been found to correlate with increased levels of EPO (Singh et al., 2016).

Often, “smoking” on clinical patient surveys does not specify a difference between cigarette smoke vs. marijuana smoking (e.g., THC; Morean et al., 2018). “Smoking” could mean any type of smoking, albeit cigarette smoke, marijuana smoking, or electronic vaporizers, etc. However, there could be very different pathophysiological effects of various types of smoking, especially at the level of AAPPs. With this in mind, we compare the literature connections among the two active chemicals in cigarette smoke (e.g., nicotine) and marijuana (tetrahydrocannabinol, referred to as THC).

Figure 8 (bottom) examines overlap and differences in ranked high residual nodes for THC and nicotine. Some of the highly ranked AAPP nodes are shared by nicotine and THC (e.g., CRH, opioid receptor, dopamine receptor, etc.)—all of these commonalities relate to the brain's reward system. That is, THC and nicotine both increase reward signaling, which makes these substances have the tendency to be addictive to their respective users.

However, there are also key differences in the AAPP rankings of the high residual nodes of nicotine and THC. For example, estrogen receptor beta was ranked 152 spots higher with respect to THC compared to nicotine, and NG-nitroarginine methyl ester was ranked 144 spots higher with respect to nicotine compared to THC. Estrogen receptor beta has a variety of physiological functions, including vasodilation, arterial dilation, cardiovascular metabolomics, and is involved in several cancers, especially breast cancer. However, estrogen receptor beta relates to cognition through its normally high expression in the hippocampus, which is part of the brain's “memory center”; loss of estrogen leads to losses in memory, expediting of brain biological aging (namely amyloid and APOE processes), which can eventually lead to Alzheimer's Disease (Foster, 2012). Nearly all of the nodes more strongly tied to THC directly correlate with decreased cognition.

Nicotine has much stronger connections to NG-nitroargimine methyl ester, as it ranks 144 spots higher with respect to nicotine compared to THC. NG-nitroargimine methyl ester is mostly associated with nitric oxide synthase (NOS) processes, which assist in respiration and oxidation pathways. Defective nitric oxide activity in the brain directly leads to decreased memory. NO has particularly strong effects on the hypothalamus, but also on the hippocampus. NO increases blood supply to the brain but as a relatively short half-life. Thus, exposure to substances that increase NO result in temporary increases in memory but can also result in longer-term depletion, which leads to impaired memory over time (Wang et al., 2018). VEGF is the other highly ranked AAPP node, which ranks 124 spots higher to nicotine compared to THC. As noted above, VEGF can actually improve cognition, and this observation has been noted in other studies examining smoking (Ugur et al., 2018). Collectively these literature results suggest why there is dichotomy in the field as to whether nicotine [from cigarette smoking] increases or decreases cognition. SemNet ranking illustrate it actually could be doing both. However, it is most likely that, over time, the small increases in cognition with nicotine use swing toward cognitive decreases, most likely resulting in an overall decrease in cognition. It could be hypothesized this is actually a habituation effect on the neurons. However, depending on individual differences in AAPP balance, there could be human sub-populations with different cognitive responses to cigarette smoking.

So what can be learned from this specific SemNet case study? Key results illustrate there are X-Y chromosomal differences in response to smoking. Thus, further examination is warranted to determine gender-specific effects. It would appear based on this initial SemNet case study that cardiovascular connections are greater to the Y chromosome while neuropeptide/neurotransmitters are stronger to the X chromosome. This would suggest that females could potentially be more at risk for cognitive effects related to smoking. Additionally, comparisons of THC and nicotine suggest that, other than shared reward/addiction commonalities, they have differently ranked AAPP connections. Nicotine has connections that illustrate a “mixed” effect on cognition, whereas the strongest THC connections all correlate with decreased cognition. This insight suggests that the definition of “smoking” on a clinical survey or epidemiological study should be framed to differentiate cigarette smoking and marijuana/THC smoking in order to better assess the differing biochemical and functional effects.

How could this initial SemNet use case be iteratively improved? The SemNet high residual nodes suggest key AAPPs that can be further investigated using more detailed SemNet searches (e.g., more specific targets, such as the high residual nodes for each ranker). As described above, much can be learned with relative ease by looking at high residual nodes from SemNet on a Venn diagram (Figure 8). However, optimization of user-specified target node input could assist in obtaining even more specific insight. For example, while several of the highly ranked nodes in the nicotine and THC example are quite specific, some are more general. Nicotine in particular has a few more general nodes that are highly ranked (e.g., “proteins,” “amino acids”), as shown in Figure 8 (bottom) compared to THC, likely because nicotine has a larger literature pool (e.g., count) than THC. Note that post-processing of non-specific terms could be done to remove terms deemed too general to be of help to a specific field, project, or study. Such a process is analogous to removing unimportant “stop words” (a, an, the, etc.) in standard text mining studies. Additionally, keep in mind this use case focused only on the AAPP node type. High residual node analysis could be repeated for any of the other 131 node types—diseases, treatments, etc.

### SemNet Applications

There are seemingly an infinite number of applications of SemNet. Clearly many fields and areas of research can benefit from leveraging a full semantic inference network of all of PubMed's 27.9 million and counting indexed abstracts. Below, we outline some of the types of literature based discovery that SemNet enables with its easy to use and adaptable structure in the popular Python language.

#### Field Maps

“Field maps” are used to visually map literature within a specific field to assess sub-topic breadth and depth, and sometimes relationships between topics. Traditionally field maps were either done by hand using expert domain knowledge or using informatics-based methods that combine text mining and expert domain knowledge (Kim et al., 2016). A key limitation of traditional field maps is they only look at sets of literature labeled as part of that field. However, the determination of “related to field” can be either narrow or subjective. There is certainly no tying of sub-topics within the field to other related pathology or physiology. In contrast, SemNet connects not only literature directly tied to the field but also beyond the field and does so in a non-subjective manner. Moreover, SemNet can rank the identified nodes, which greatly enhances the level of information and context provided.

#### The Ultimate Systematic Review and Meta-Analysis

Systematic reviews and meta-analyses, despite rigorous methodology recommended by Cochrane Systematic Reviews, can suffer from selection bias (Graves et al., 1996). SemNet is a great platform for assisting a user that wants to perform the ultimate systematic review. SemNet enables target-to-source node relationships to be visualized in a way that extends beyond a few key terms. Again, SemNet's ranking system enables prioritization. Having a better and comprehensive understanding of node and metapath structure enables comprehensive and less biased selection criteria. With the understanding imparted by SemNet to optimize selection criteria and expand article selection, articles can then have their internal quantitative data curated in bulk (Mitchell et al., 2015) and statistically analyzed.

SemNet thus can clearly improve the all-important initial article identification and inclusion required for a systematic review and meta-analysis. However, SemNet, which is based on text analysis, cannot yet perform the statistical calculations directly on articles' quantitative figure and table data. However, future updates to SemNet to assign rankings to actual metapath relationships could make SemNet a standalone tool for meta-analysis.

#### Therapeutic Identification and Drug Repurposing

Semantic inference networks have primarily been previously used for drug re-purposing (Kilicoglu, 2017). However, previous tools have not been as comprehensive and/or have been difficult to operate for the average user. The ease and flexibility of SemNet and its implementation in the popular Python language make it more accessible and adoptable to a greater number of users. SemNet's use of SemMedDB enables therapeutic identification based on disease, symptom, risk factor, functional measure, or any number of multi-scalar pathological or physiological nodes. SemNet is not limited to merely examining nodes related to “omics” (gene or protein expression), which greatly expands its capabilities to find potential therapeutics or targets based on multiple, multi-scalar targets.

#### Epidemiology and Risk Profiling

Due to the same features outlined above for drug identification, SemNet can also be utilized to perform more comprehensive assessments of epidemiology and risk profiling. Metapaths tied to a given set of cohort characteristics set as target nodes can be used to explore relevant multi-scalar physiological, pathological, and therapeutic nodes, which can be used to develop conceptual patient risk profiles. The case use example comparing X and Y chromosomes, as shown in Figure 8 (top), illustrates how differences in risk or etiology between males or females could be compared based on literature relationships.

### Limitations and Future Directions

As previously noted, clustering of biomedical concepts using SemNet does not directly represent biological similarity, but similarity in patterns of connections to the target nodes found by SemRep. Thus, the resulting concept network is only as good as the literature it represents. The National Library of Medicine's SemMedDB is updated regularly, which does allow for reconstruction of any analyses using new journal article abstracts. However, clearly SemRep cannot identify relationships between nodes if those relationships, albeit known or unknown to the user, do not exist in the literature. However, examination of nodes and especially DWPC for a particular target node does allow the user to determine potential “holes,” or areas of low connectivity in the literature, where future research could be extremely valuable. Thus, even the lack of expected underlying metapaths could justify the need for research on a given topic, relationship, or hypothesis.

Like any given model, SemNet is dependent on the amount and quality of the data of which it is comprised–the articles in PubMed, or more specifically, SemMedDB. It is no secret that judging the quality of a scientific article is subjective and the variance of literature quality within PubMed is considered vast by most researchers. Thus, some researchers or potential SemNet users may worry if perceptually lesser quality research publications are included. Large sample size likely drowns most potential issues caused by a smaller percentage of presumed lesser quality publications. Nonetheless, the perceived quality of the input data set could be controlled if so desired, albeit by impact factor, journal name, etc. by altering queries to SemMedDB. However, care would need to be taken to not overly bias the input data sources, and in doing so, lose the benefit of agnostic exploration provided by a large-scale semantic inference network of a comprehensive scientific body of literature.

While the presented ULARA ranking algorithm (Klementiev et al., 2007) has been adopted by the text mining field and appears excellent for general purpose use in SemNet, the ranking module of SemNet could be swapped for future more advanced methods or other existing rankers that have a more project-specific goal. In combination with ULARA, additional features beyond count, DWPC, and HeteSim could be added or swapped into SemNet to help improve rankings as feature methods are improved in the future. Furthermore, future addition of relationship weighting features (as mentioned under the Systematic Review and Meta-Analysis section) would improve context and function using the SemNet platform.

There is still much to learn about how heterogeneous semantic networks “behave.” For example, there is an infinite number of sensitivity analyses that could be performed in the future to examine how corpus size, node counts, and degree of connectivity impacts the overall network results and especially SemNet projected rankings. This information could be used to help further optimize the platform and better assist in results interpretation.

### Conclusion

In conclusion, a semantic network created using the text of biomedical abstracts can be effectively used to identify, rank, and cluster relevant concepts to a user-specified target of interest. This allows the user to quickly develop an up-to-date model of a topic by navigating the biomedical concept graph. SemNet is an excellent starting point for virtually any biomedical concept, project, or data aggregation study, which wishes to utilize literature based discovery. Specifically, SemNet could be of great value for identifying relationships that impact the ranking of specific diseases risk factors, multi-scalar pathophysiology, or therapeutic identification, including off-label drug re-purposing. SemNet can highlight both areas of high literature connectivity and as well as literature “holes” (e.g., areas where there are few publications or connections). In summary, the ability to easily connect and visualize relationships using all of PubMed to examine targets of interest is a valuable asset and major step forward for literature based discovery.

We have compiled the code into a Python package that we call SemNet. The package is open-source, along with the network data adapted from the National Library of Medicine. Additionally, detailed online documentation has been assembled for the SemNet software. Download on GitHub.

## Author Contributions

AS: framing of study, code development and implementation, statistical analysis, results interpretation, and critical review of content. CM: framing of study, project oversight, results interpretation, drafting of final manuscript, and critical review of content.

### Conflict of Interest Statement

The authors declare that the research was conducted in the absence of any commercial or financial relationships that could be construed as a potential conflict of interest.
